# MicroRNA‐203–mediated inhibition of doublecortin underpins cardioprotection conferred by sevoflurane in rats after myocardial ischaemia‐reperfusion injury

**DOI:** 10.1111/jcmm.15566

**Published:** 2020-08-11

**Authors:** Jian Tan, Zhiguo Wu, Jun Liu, Wenting Zhang, Wanqiu Yuan, Hong Peng

**Affiliations:** ^1^ Department of Anesthesiology Pingxiang People's Hospital of Southern Medical University Pingxiang P. R. China; ^2^ Department of Obstetrics Pingxiang Maternity and Child Health Hospital Pingxiang P. R. China

**Keywords:** doublecortin, inflammation, microRNA‐203, myocardial ischaemia‐reperfusion injury, oxidative stress, sevoflurane

## Abstract

Myocardial ischaemia‐reperfusion (I/R) injury is a serious illness with high morbidity and mortality. Mounting evidence indicates the utility of sevoflurane (SEV) in the treatment of myocardial I/R injury. This study aimed to explore the molecular mechanisms underlying the protective action of SEV against myocardial I/R injury. A rat model of myocardial I/R injury was established, and I/R rats were treated with different concentrations of SEV. MicroRNA‐203 (miR‐203) and doublecortin (DCX) expression levels were determined using reverse transcription‐quantitative polymerase chain reaction. Putative target relationship between miR‐203 and DCX was explored using dual‐luciferase reporter gene assay and RNA‐binding protein immunoprecipitation assay. Ischaemia‐reperfusion rats were treated with SEV, miR‐203 antagomir or sh‐DCX, followed by determination of oxidative stress‐ and inflammation‐related factor levels using nitrite and enzyme‐linked immunosorbent assays, and that of apoptosis‐related factors using Western blot analysis. The apoptotic rate of myocardial tissues was determined using TdT‐mediated dUTP‐biotin nick end labeling (TUNEL) staining, and the infract area was evaluated using triphenyltetrazolium chloride staining. The results showed miR‐203 was poorly expressed and DCX was highly expressed in myocardial tissues of I/R rats. Sevoflurane was found to elevate miR‐203, and miR‐203, in turn, could target and reduce DCX expression. Sevoflurane, miR‐203 overexpression or DCX silencing resulted in declined oxidative stress, inflammation, apoptosis and infarct area, ultimately alleviating myocardial I/R injury. Collectively, these findings showed that SEV‐activated miR‐203 exhibited suppressive effects on myocardial I/R injury in rats and highlighted the SEV/miR‐203/DCX axis as a promising therapeutic target for myocardial I/R injury management.

## 1 INTRODUCTION

Myocardial ischaemia‐reperfusion (I/R) injury is a vital factor involved in myocardial infarction, causing damage to cardiac tissues including oxidative stress, inflammation and cell apoptosis.^1^ The mechanisms underlying I/R injury are extraordinarily complex, highly interconnected and influenced by multiple factors such as platelet function, antiplatelet drugs, coronary and diabetic diseases.^2^ More recently, studies have investigated and validated the value of sevoflurane (SEV) in the treatment of myocardial I/R injury.^3^, ^4^ Sevoflurane, an inhalational anaesthetic, has been identified to mediate multiple processes including oxidative stress, mitochondrial damage and neuronal apoptosis.^5^ It has been reported that the action of SEV in myocardial I/R injury is mediated by microRNA‐135b‐5p.^6^ This suggests that SEV may regulate other microRNAs (miRNAs) as molecular mechanisms underlying its effects against myocardial I/R injury.

MicroRNAs are small non‐coding RNAs and understood to be key regulators of events leading to the progression of I/R.^7^ In particular, a protective effect of microRNA‐203 (miR‐203) against I/R injury after total knee arthroplasty has been reported in mice.^8^ Also, miR‐203 down‐regulation has been noted to exacerbate myocardial I/R by increasing cardiomyocyte inflammation and myocardial injury.^9^ Notably, SEV has been found inhibit the progression of breast cancer via an up‐regulation of miR‐203.^10^ Doublecortin (DCX) is an essential mediator in the process of neuronal migration and cortical layering during brain development ^11^ and is a microtubule‐binding protein produced during neurogenesis.^12^ It has been associated with ischaemia and has been found to play a key role in mediating global brain ischaemia.^13^ Changes in DCX expression in the ischaemic area have been reported as a marker of neurological functional recovery in rats caused by the Gualou Guizhi decoction.^14^ Importantly, bioinformatics analysis available on https://cm.jefferson.edu/rna22/Interactive/ revealed DCX as a potential target gene of miR‐203. Based on existing evidence, it may be inferred that SEV exerts cardiac protective effects through the regulation miR‐203. However, the specific nature of putative interactions between SEV, miR‐203 and DCX in rats with myocardial I/R injury has not been investigated. Therefore, the present study was designed with a goal of expanding the understanding of molecular mechanisms underlying the protective role of SEV in myocardial I/R injury and thus provide a basis for novel target discovery in this context.

## 2 MATERIAL AND METHODS

### 2.1 Ethics statement

The study protocol was approved by the animal committee of Pingxiang People's Hospital of Southern Medical University. All procedures were performed in strict accordance with the recommendations of the Guide for the Care and Use of Laboratory Animals and all efforts were made to minimize the numbers and suffering of the included animals.

### 2.2 Chemicals and reagents

Pentobarbital sodium (Sigma‐Aldrich, St. Louis, MO, USA); malondialdehyde (MDA) (A003‐1‐2), superoxide dismutase (SOD) (A001‐3‐2) and glutathione (GSH) (A006‐2‐1) (Nanjing Jiancheng Bioengineering Institute, Nanjing, China); TRIzol reagents (Invitrogen, Waltham, Massachusetts, USA); miR‐203 antagomir, antagomir negative control (NC), lentiviral vector expressing shRNA targeting DCX or scrambled shRNA (GenePharma, Shanghai, China); TaqMan MicroRNA Assays Reverse Transcription Primer (4427975; Applied Biosystems, Waltham, Massachusetts, USA); magnetic beads conjunct with anti‐Argonaut‐2 (Ago‐2) antibody (BMFA‐1; Biomarker Technologies, Rohnert Park, CA, USA); pMIR‐reporter containing the DCX 3ʹuntranslated region (3ʹUTR) (Promega, Madison, WI, USA); Dual‐Luciferase Reporter Assay System kit (Promega); RNasin (Takara, Japan); protease inhibitor cocktails (B14001a; Roche, Basel, Switzerland); bicinchoninic acid assay (BCA) kit (P0011; Beyotime Biotechnology, Wuhan, China); anti‐glyceraldehyde‐3‐phosphate dehydrogenase (GAPDH) antibody (5174s, 1:1000; Cell Signaling Technology, Danvers, MA, USA); anti‐Cyt‐3 antibody (11940s, 1:1000, Cell Signaling Technology); anti‐cleaved caspase‐3 antibody (9661s, 1:1000, Cell Signaling Technology); anti‐tumour necrosis factor alpha (TNF‐α) antibody (11948s, 1:200; Cell Signaling Technology); anti‐interleukin (IL)‐6 antibody (12912s, 1:200; Cell Signaling Technology); anti‐IL‐1β antibody (12703s, 1:1000; Cell Signaling Technology); polyvinylidene fluoride (PVDF) membrane (Millipore, Billerica, MA, USA); ELISA kits for IL‐1β, TNF‐α and IL‐6 (Wuhan Moshake, Wuhan, China); Nitrite kit (Nanjing Jiancheng Bioengineering Institute); DeadEnd Fluorometric TUNEL System kit (Promega); triphenyltetrazolium chloride solution (B011072A; Chengdu Best Reagent Co., Ltd., Chengdu, Sichuan, China; pH = 7.4); SEV (Y0001046‐1EA), glutaraldehyde (G5882), acetone (650501) and TritonX‐100 (T9284) all from Sigma‐Aldrich; PBS (C10010500BT; Gibco, Gaithersburg, MD, USA); haematoxylin‐eosin (G1120; Solarbio, Beijing, China); azo blue solution (DK0001; Solarbio); ethanol (Beijing Chemical Works, Beijing, China); uranyl acetate‐lead citrate (GZ02618; Electron Microscopy China, Nanjing, Jiangsu, China); lysis buffer (P0013B), paraformaldehyde (P0099), TdT reaction solution (D7076) and 4′,6‐diamidino‐2‐phenylindole (DAPI) (C1002) all from Beyotime Biotechnology; Horseradish Peroxidase (HRP)‐labelled secondary antibody (Santa Cruz Biotechnology, CA, USA); 10% SDS‐PAGE (Shanghai Willget Biotech, Shanghai, China); magnetic beads conjunct with anti‐immunoglobulin G (IgG) (BMSR; BioMag, Wuxi, Jiangsu, China); and enhanced chemiluminescence (ECL) reagents (WBULS0100, Millipore, MIT, USA).

### 2.3 Rat models of myocardial I/R injury

Myocardial I/R injury was induced in 180 adult male Sprague Dawley (SD) rats (Vital River Laboratory Animal Technology Co., Ltd., Beijing, China) aged 7‐8 weeks and weighing (250 ± 50) g. In brief, the rats were fasted for 12 hours with free access to water and then anaesthetized by intraperitoneal injection of 50 mg/kg 3% pentobarbital sodium. The rat heart was exposed under sterile conditions through a left thoracotomy in the fourth intercostal space. Ischaemia was achieved by ligation of the left anterior descending coronary artery (LAD) and confirmed by sinus tachycardia (ST) segment elevation in the electrocardiogram. After a 30‐minute period of occlusion of LAD, a 120‐minute period reperfusion was performed. Sham‐operated rats (n = 20) underwent a similar surgical procedure without LAD ligation. The criteria for successful preparation of the I/R model were as follows: under ischaemic conditions, myocardial tissues were seen as pale and cyanotic along with obvious ST segment elevation or higher T wave as observed by Electrocardiogram (ECG), whereas after reperfusion, the myocardial tissues in the ischaemic region turned red, while the elevation of ST segment that occurred under ischaemic conditions decreased by more than 50%.

### 2.4 SEV exposure and knockdown of cardiac miR‐21 and DCX expression in vivo

Among 200 rats to be I/R modelled, 20 rats received intramyocardial injections of 2 μg normal saline five times, 24 hours before I/R modelling (named I/R group); 60 rats were exposed to 1%SEV, 2%SEV and 4%SEV, respectively, for 5 minutes during reperfusion (named 1%SEV, 2%SEV and 4%SEV groups); 20 rats received intramyocardial injection of 2 μg miR‐203 antagomir^15^ five times, 24 hours before I/R modelling (named miR‐203 antagomir group); 20 rats received intramyocardial injection of 2 μg antagomir‐NC five times, 24 hours before I/R modelling (named antagomir‐NC group); 20 rats received intramyocardial injection of 2 μg miR‐203 antagomir five times, 24 hours before I/R modelling and were exposed to 4%SEV for 5 minutes during reperfusion (named SEV + miR‐203 antagomir group); 20 rats received intramyocardial injection of 2 μg lentiviral vector expressing shRNA targeting DCX five times, 24 hours before I/R modelling (named sh‐DCX group); 20 rats received intramyocardial injection of 2 μg lentiviral vector expressing scrambled shRNA five times, 24 hours before I/R modelling (named sh‐NC group); and 20 rats received intramyocardial injection of 2 μg lentiviral vector expressing shRNA targeting DCX and 2 μg miR‐203 antagomir both five times, 24 hours before I/R modelling.

### 2.5 Hemodynamic measurement

After 4 hours of reperfusion, rats were anaesthetized by intraperitoneal injections of 3% pentobarbital sodium and then connected to a ventilator. The left common carotid artery was isolated, inserted into the left ventricle and connected to a pressure transducer. The left ventricular systolic pressure (LVSP) and left ventricular end‐diastolic pressure (LVEDP) were each measured by a carrier amplifier. Thereafter, the maximum rate of left ventricular pressure rise [LVdp. (d*t*_max_)^−1^] and the maximum rate of left ventricular pressure drop [LVdp. (d*t*_min_)^−1^] were calculated using a differentiator.

### 2.6 Serum myocardial enzymogram determination

After hemodynamic measurement, 2 mL of blood was collected from the abdominal aorta. After 30 minutes of concentration at room temperature, the blood was centrifuged at 2515 *g* at 4°C for 15 minutes. The supernatant serum was collected, and creatine kinase isoenzyme (CK‐MB) and lactate dehydrogenase (LDH) activity was each measured using an automatic biochemical analyzer (Model 170A; Hitachi, Tokyo, Japan), while cardiac troponin T (cTn‐T) was detected using an ELISA kit.

### 2.7 Enzyme‐linked immunosorbent assay

The blood obtained from rat myocardial tissues was allowed to stand at room temperature and stored at 4°C overnight. The clear serum of the supernatant was collected through centrifugation at 3500× *g*, and frozen at −80°C. The levels of IL‐1β, IL‐6 and TNF‐α in serum were measured using murine IL‐1β, IL‐6 and TNF‐α ELISA kits (Wuhan MSKBio Co., Ltd., Wuhan, Hubei, China), respectively, in accordance with manufacturer's protocol. In addition, cells were cultured for 24 hours, and the cell culture medium was centrifuged at 1000× *g* for 10 minutes at room temperature to collect the cell supernatant. IL‐1β, TNF‐α and IL‐6 in the cell culture medium were measured as above and standard curves were plotted.

### 2.8 Triphenyltetrazolium chloride staining

Six rats from each experimental group were euthanatized, and the hearts were excised. After perfusion of normal saline through the carotid artery, the heart was injected with 0.5 mL of 1.5% azo blue solution. The heart tissue sections (1‐mm thick) were obtained and incubated in 2% triphenyltetrazolium chloride (TTC) solution at 37°C for 30 minutes to discriminate non‐infarct and infarct areas, where myocardium in the ischaemic and non‐infarct risk zone was stained in red while infarct myocardium was greyish white. After isolation of the ischaemic and infarct myocardium, the wet weights of each were measured using an electronic scale, and computations were made using the formulae: Area‐at‐risk/left ventricle (AAR/LV) × 100% = myocardial ischaemic area, and infarct area/AAR (IA/AAR) × 100% = myocardial infarct area.

### 2.9 Rat cardiac tissue specimen collection, haematoxylin‐eosin staining and transmission electron microscope (JOEL1010) observation

The fourth rat from each experimental group received intraperitoneal injections of 120 to 150mg/kg 3% pentobarbital sodium and underwent thoracotomy followed by LAD ligation. The rats then received 1 mL 1% patent blue injection through the internal jugular vein, and their hearts were excised and fixed with 4% paraformaldehyde for 24 hours. The heart paraffin‐embedded and prepared into 5 μm sections by cryosection using an ultramicrotome (Olympus, Shinjuku, Japan). Paraffin‐embedded sections from six rats of each group were haematoxylin‐eosin stained and cryosectioned for transmission electron microscope (JOEL1010) observation.

### 2.10 Nitrite assay

The nitrite in the supernatant was used as an indicator of nitric oxide (NO) production and determined using an oxidized nitrite kit according to the manufacturer's instructions. In brief, the supernatant was sequentially mixed with an equal volume of Griess reagent I and Griess reagent II, followed by measurement of the optical density (OD) value at 540 nm using a Smart‐Spec Plus spectrophotometer (Bio‐Rad, Hercules, CA, USA), and the standard curve was plotted with 0 ‐ 100 μm nitrite sodium. The sodium nitrite concentration was calculated based on the OD values at 540 nm.

### 2.11 Determination of mitochondrial SOD activity and reduced GSH and MDA content

The rat myocardial tissues were added with 1 mL of PBS, and supernatant was obtained by centrifugation at 12 000× *g* for 10 minutes at 4°C. Malondialdehyde, SOD and GSH activities in the myocardial tissues were measured using MDA, SOD and GSH assay kits (Nanjing Jiancheng Bioengineering Institute), respectively. Cells in the logarithmic growth phase were seeded into a 6‐well plate at a density of 2 × 10^6^ cells/mL, with 2 mL of culture medium in each well. After 48 hours of culture, the culture medium was discarded, and the above‐mentioned assay kits were used to assess the concentration of SOD, MDA and GSH, respectively, in the cells.

### 2.12 RNA‐binding protein immunoprecipitation assay

The myocardial cell line H9C2 (Sigma‐Aldrich, St. Louis, MO, USA) was lysed using lysis buffer (25 mmol/L Tris‐HCl pH = 7.4, 150 mmol/L NaCl, 0.5% Nonidet P‐40, 2 mmol/L ethylenediaminetetraacetic acid, 1 mmol/L NaF and 0.5 mmol/L dithiothreitol) containing RNasin and protease inhibitor. Centrifugation was performed at 12 000× *g* for 30 minutes, and the supernatant was collected. Then, anti‐human Ago‐2 magnetic beads were added, followed by anti‐IgG magnetic added as control. Incubation was done at 4°C for 4 hours, and the beads were washed three times with wash buffer (50 mmol/L Tris‐HCl, 300 mmol/L NaCl pH = 7.4, 1 mmol/L MgCl_2_, 0.1% Nonidet P‐40). RNA was extracted from the magnetic beads using TRIzol, and the expression levels of miR‐203 and DCX were measured using reverse transcription‐quantitative polymerase chain reaction (RT‐qPCR).

### 2.13 TUNEL staining

In brief, the cells were fixed with 4% paraformaldehyde at 4°C for 25 minutes, ruptured with 0.2% Triton X‐100 for 5 minutes and balanced with 100 μL equilibrium solution per well at room temperature for 5‐10 minutes. The TdT reaction solution was prepared once the cells were balanced. The components in each well included 45 μL equilibration buffer, 5 μL nucleotide mixture and 1 μL TdT. Then, the equilibrium solution was aspirated, 50 μL of TdT reaction solution was added to each well, and the reaction was carried out at 37°C for 1 hour. Thereafter, 50 μL of 2× standard sodium citrate was added to terminate the reaction, and the mixture was allowed to stand at room temperature for 15 minutes in the dark. Then, 6‐diamidino‐2‐phenylindole (1 μL/mL) was added and incubated with cells at room temperature for 15 minutes in the dark, followed by addition of anti‐fade solution (Cat#57461; Molecular Probe, Eugene, OR, USA). Cells in different fields of view were photographed under a fluorescence microscope (IX71‐F22FL/DIC; Olympus) at 488 and 405 nm, respectively, with at least 200 cells present in each field. Five fields were randomly selected for each sample, and the number of cells was counted for apoptosis detection, followed by statistical analysis.

### 2.14 Dual‐luciferase reporter gene assay

A miR‐203‐mRNA prediction analysis was performed using a web‐based resource (https://cm.jefferson.edu/rna22/Interactive/). Thereafter, artificially synthesized DCX 3ʹUTR gene fragment containing miR‐203 binding sites was constructed into pMIR‐reporter. The complementary sequence mutant (MUT) site of the seed sequence was designed on the DCX wild‐type (WT) and constructed into the pMIR‐reporter. The desired luciferase reporter plasmids WT and MUT were cotransfected into rat H9C2 cardiomyocytes with miR‐203 mimic, respectively. After 48 hours of transfection, the cells were harvested and lysed, and luciferase activity was measured using a Dual‐Luciferase Reporter Assay System.

### 2.15 Reverse transcription‐quantitative polymerase chain reaction

Total RNA was extracted using a TRIzol kit and reverse transcribed into cDNA using a TaqMan MicroRNA Assays Reverse Transcription Primer, according to standard instructions. 5 μL of the cDNA products was used as the template for PCR amplification. The PCR reaction volume was 25 μL: 5 μL of reverse transcription products, 13 μL of 2× QuantiTect SYBR Green RT‐PCR Master Mix, 0.5 μL of upstream primer and downstream primer (10 μmol/μL) each and 6 μL of DNAase‐free water. The reaction conditions were as follows: 95°C for 5 minutes (one cycle), 95°C for 20 seconds, 60°C for 1 minute and 72°C for 30 seconds (40 cycle). The U6 gene was considered as an internal reference for miR‐203, and GAPDH was used as an internal reference for the other genes. The primers are depicted in Table 1. The relative expression levels of the target genes were calculated using the 2^−ΔΔCT^ method.

**TABLE 1:** Primer sequence for RT‐qPCR

Name	Primer sequence
miR‐203	F: GGGGTGAAATGTTTAGGAC
	R: CAGTGCGTGTCGTGGAGT
DCX	F: AGCCAAGAGCCCTGGTCCTAT
	R: TGGAGGTTCCGTTTGCTGAGT
U6	F: CTCGCTTCGGCAGCACA
	R: AACGCTTCACGAATTTGCGT
GAPDH	F: GCACCGTCAAGGCTGAGAAC
	R: GGATCTCGCTCCTGGAAGATG

Abbreviations: DCX, doublecortin; F, forward; GAPDH, glyceraldehyde‐3‐phosphate dehydrogenase; miR‐203, microRNA‐203; R, reverse; RT‐qPCR, reverse transcription‐quantitative polymerase chain reaction.

### 2.16 Western blot analysis

Rat hearts from each group were collected immediately following myocardial I/R, added with lysis buffer, shaken on a vortex and centrifuged at 6037 *g* for 30 minutes at 4°C to remove tissues or cell debris. The supernatant was obtained and the total protein concentration was measured using a BCA kit. Thereafter, 50 μg of proteins was dissolved in 2× sodium dodecyl sulphate (SDS) loading buffer and boiled at 100°CC for 5 minutes. Each sample was then subjected to 10% SDS‐polyacrylamide gel electrophoresis and the proteins were transferred to PVDF membranes by the wet transfer method. The membrane was blocked with 5% skim milk powder at room temperature for 1 hour and then probed with diluted primary antibodies to GAPDH (1:1000), Cyt‐3 (1:1000), cleaved caspase‐3 (1:1000), TNF‐α (1:200), IL‐6 (1:200) and IL‐1β (1:1000). The membrane was then re‐probed with horseradish peroxidase‐labelled secondary antibody for 1 hour. An ECL fluorescence detection kit was employed for colour development, and imaging was performed using a gel imager. The proteins were photographed using a Bio‐Rad Image Analysis System (BIO‐RAD, Hercules, CA, USA) and analysed with Quantity One v4.6.2 software. The relative protein level was defined as the grey value of the corresponding protein band/the grey value of the GAPDH protein band.

### 2.17 Statistical analysis

The Statistic Package for Social Science 21.0 statistical software (IBM Corp, Armonk, NY, USA) was used for performing statistical analysis. Measurement data were expressed as mean ± SD. For unpaired data with normal distribution and homogeneity of variance, comparisons between two groups were analysed by independent sample *t* test and comparisons between multiple groups were performed using one‐way ANOVA with Tukey's post hoc tests. A value of *P* < 0.05 was considered statistically significant.

## 3 RESULTS

### 3.1 SEV up‐regulated miR‐203 to improve the histological morphology and reduce apoptotic rate and infarct area, in rats with myocardial I/R injury

**FIGURE 1:**
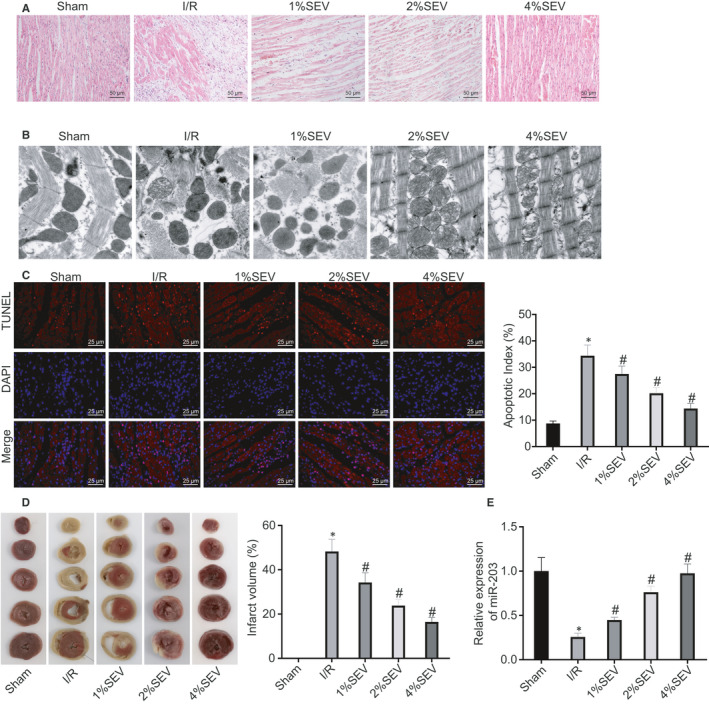
Sevoflurane (SEV) activates microRNA‐203 (miR‐203) and suppresses the apoptotic rate and infarct size in rat with myocardial ischaemia‐reperfusion (I/R) injury. Sham‐operated rats were used as controls, and I/R rats were treated with 1% SEV, 2% SEV or 4% SEV or untreated. A, Representative histological morphology of myocardial tissues observed by haematoxylin‐eosin staining (×200). B, Representative ultrastructure of myocardial tissues observed by transmission electron microscope (×3000). C, Representative image of myocardial apoptosis determined by TUNEL staining (×400) and apoptotic rates. D, Infarct area observed by triphenyltetrazolium chloride staining and infarct volume. E, The expression of miR‐203 in rat myocardial tissues determined by reverse transcription‐quantitative polymerase chain reaction, normalized to U6. *P < 0.05 vs rats receiving sham operation; ^#^ *P* < 0.05 vs rats with myocardial I/R injury by one‐way ANOVA, followed by Tukey's post hoc tests. Results were shown as mean ± SD from three technical replicates (n = 6‐8)

In order to study the effects of SEV on myocardial I/R injury, a rat model of myocardial I/R injury was developed on SD rats and SEV treatment was provided. Haematoxylin‐eosin staining demonstrated that myocardial fibrosis disorder, degeneration, rupture and myocardial cell oedema were severe in rats with myocardial I/R injury. However, after SEV treatment, the myocardial fibres were arranged more neatly and myocardial cell oedema was found reduced in a dose‐dependent manner (Figure 1A). As shown in Figure 1B, the myocardial tissues were irregularly dissolved, the mitochondrial vacuoles were swollen, and disordered arrangement was noted in I/R rats. After I/R rats were treated with SEV, the myocardial sarcomeres were seen as relatively clearer, the dissolved myofilaments were reduced, and the mitochondrial microcavitation was found attenuated in a dose‐dependent manner (Figure 1B). Moreover, I/R treatment led to markedly elevated apoptotic rate (Figure 1C) and increased the infarct size of myocardial tissues (Figure 1D) in rats, which was decreased upon SEV treatment in a dose‐dependent manner. Reverse transcription‐quantitative polymerase chain reaction was performed to assess whether SEV could up‐regulate miR‐203 in rats with myocardial I/R injury. It was observed that miR‐203 expression decreased remarkably in rats with myocardial I/R injury and increased in I/R rats treated with SEV, and the effect of SEV appeared dose‐dependent (Figure 1E). These results indicated that SEV could up‐regulate miR‐203 to decrease the apoptotic rate and infarct size in rats with myocardial I/R injury.

### 3.2 Up‐regulation of miR‐203 by SEV improved oxidative stress and inflammatory response in rats with myocardial I/R injury

**FIGURE 2:**
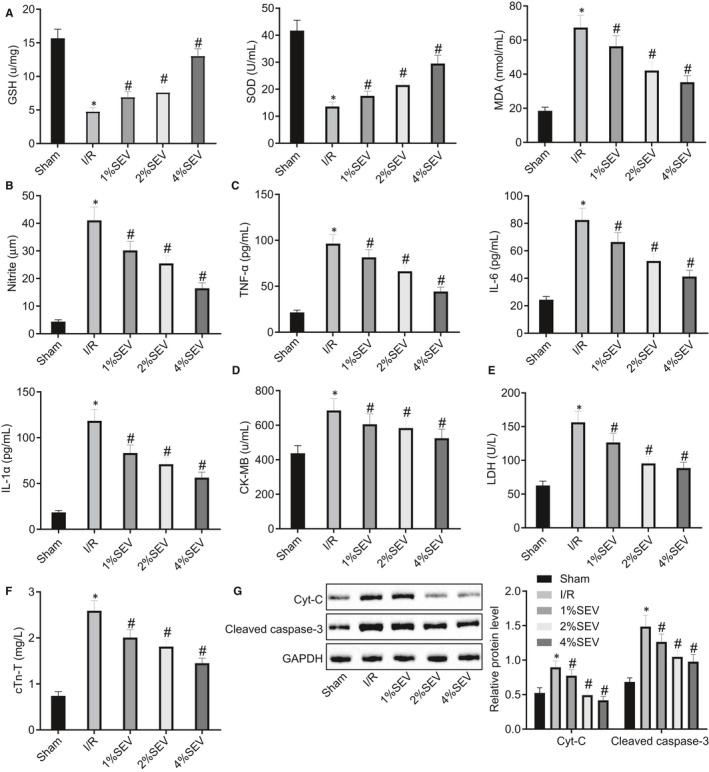
Oxidative stress and inflammation in rats with myocardial ischaemia‐reperfusion (I/R) injury are repressed by sevoflurane (SEV) exposure before modelling in a dose‐dependent manner. Sham‐operated rats were used as controls, and I/R rats were treated with 1% SEV, 2% SEV or 4% SEV or untreated. A, Expression of malondialdehyde (MDA), glutathione (GSH) and superoxide dismutase (SOD) in rat myocardial tissues determined by ELISA. B, Nitric oxide level in rat serum determined by Nitrite assay. C, Expression of tumour necrosis factor alpha (TNF‐α), interleukin (IL)‐6 and IL‐1β in rat serum determined by ELISA. D‐F, Expression of creatine kinase isoenzyme (CK‐MB), lactate dehydrogenase (LDH) and cTn‐T in rat serum determined by ELISA. G, Representative Western blots and their quantification of Cyt‐c and cleaved caspase‐3, normalized to glyceraldehyde‐3‐phosphate dehydrogenase (GAPDH). *P < 0.05 vs sham‐operated rats; ^#^ *P* < 0.05 vs I/R rats by one‐way ANOVA, followed by Tukey's post hoc tests. Results were shown as mean ± SD from three technical replicates (n = 6‐8)

Hemodynamic assessment of the rats with myocardial I/R injury illustrated that LVSP, +d*p*/d*t*_max_ and −d*p*/d*t*_min_ were decreased while LVEDP was increased markedly, which was reversed upon treatment with SEV (Table 2). We next determined the levels of oxidative stress markers (MDA, NO, GSH and SOD) in the rat myocardial tissues, and pro‐inflammatory factors (TNF‐α, IL‐6 and IL‐1β) and myocardial enzymes (CK‐MB, LDH and cTn‐T) in rat serum. As shown in Figure 2A‐F, following I/R model establishment, the levels of MDA, NO, TNF‐α, IL‐6, IL‐1β, CK‐MB, LDH and cTn‐T were elevated, but GSH and SOD levels declined, which was reversed by SEV exposure in a dose‐dependent manner. In addition, Western blot analysis revealed that the expression of apoptosis‐related factors (Cyt‐c and cleaved caspase‐3) in myocardial tissues of I/R rats was higher than that in sham‐operated rats, and SEV reversed these trends in I/R rats in a dose‐dependent manner (Figure 2G). Collectively, these data showed that SEV could improve oxidative stress and inflammation in rats with myocardial I/R injury in a dose‐dependent manner, with rats treated with 4% SEV showing the most obvious changes. Therefore, in subsequent experiments, 4% SEV was used as the experimental condition.

**TABLE 2:** Effect of SEV on hemodynamics of rats with myocardial I/R injury

Group	LVSP (mm Hg)	LVEDP (mm Hg)	+d*p*/d*t*_max_	−d*p*/d*t*_min_
			(Kmm Hg/s)	(Kmm Hg/s)
Sham	76.28 ± 6.48	8.38 ± 0.68	1.96 ± 0.21	−1.33 ± 0.13
I/R	50.63 ± 4.38*	22.58 ± 2.15*	0.83 ± 0.06*	−0.58 ± 0.04*
1% SEV	57.36 ± 4.03^#^	20.58 ± 1.94^#^	0.96 ± 0.09^#^	−0.69 ± 0.07^#^
2% SEV	61.58 ± 6.25^#^	18.38 ± 1.69^#^	0.91 ± 0.08^#^	−0.77 ± 0.06^#^
4% SEV	71.06 ± 7.26^#^	17.18 ± 1.58^#^	1.68 ± 0.15^#^	−0.91 ± 0.09^#^

Note

Results were shown as mean ± SD from three technical repeats (n = 6‐8).

Abbreviations: I/R, ischaemia‐reperfusion; LVEDP, left ventricular end‐diastolic pressure; LVSP, left ventricular systolic pressure; SEV, sevoflurane.

* *P* < 0.05 vs sham‐operated rats.

^#^ *P* < 0.05 vs I/R rats.

### 3.3 Up‐regulation of miR‐203 by SEV protected rats against myocardial I/R injury

**FIGURE 3:**
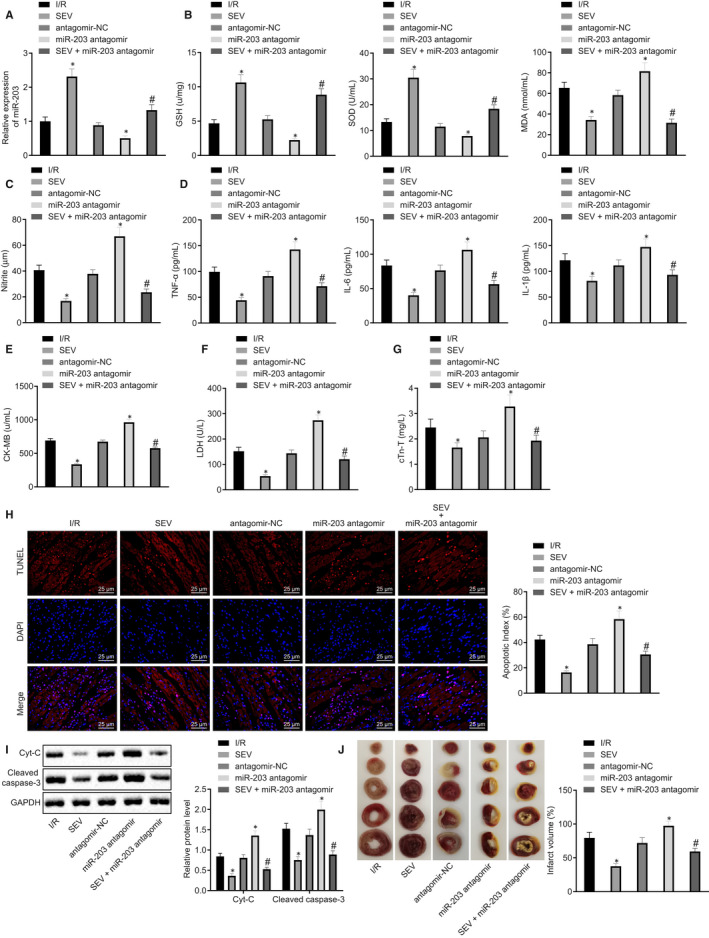
Myocardial ischaemia‐reperfusion (I/R) injury in rats is ameliorated by sevoflurane (SEV) through up‐regulation of microRNA‐203 (miR‐203). I/R rats were treated with SEV, antagomir‐negative control (NC), miR‐203 antagomir or SEV + miR‐203 antagomir or untreated. A, Expression of miR‐203 in rat myocardial tissues determined by reverse transcription‐quantitative polymerase chain reaction normalized to U6. B, Expression of malondialdehyde (MDA), glutathione (GSH) and superoxide dismutase (SOD) in rat myocardial tissues measured by ELISA. C, Nitric oxide level in rat serum determined by Nitrite assay. D, Levels of tumour necrosis factor alpha (TNF‐α), interleukin (IL)‐6 and IL‐1β in rat serum measured by ELISA. E, Level of creatine kinase isoenzyme (CK‐MB) in rat serum determined by ELISA. F, Level of lactate dehydrogenase (LDH) in rat serum measured by ELISA. G, Level of cTn‐T in rat serum determined by ELISA. H, Representative myocardial apoptosis determined by TUNEL staining (×400) and apoptotic rates. I, Representative Western blots and their quantification of Cyt‐c and cleaved caspase‐3 in rat myocardial tissues, normalized to glyceraldehyde‐3‐phosphate dehydrogenase (GAPDH). J, Infarct size determined by triphenyltetrazolium chloride staining and infarct volume. *P < 0.05 vs I/R rats; ^#^ *P* < 0.05 vs I/R rats treated by SEV by one‐way ANOVA, followed by Tukey's post hoc tests. Results were shown as mean ± SD from three technical replicates (n = 6‐8)

To further investigate whether SEV affected myocardial oxidative stress and inflammation by up‐regulating miR‐203, RT‐qPCR was used to determine the inhibitory effects of miR‐203 antagomir on miR‐203. The results indicated that miR‐203 expression in the I/R rats was enhanced by SEV treatment, which was abrogated by miR‐203 antagomir. Moreover, miR‐203 expression levels in I/R rats were decreased by miR‐203 antagomir (Figure 3A). LVSP, +d*p*/d*t*_max_ and −d*p*/d*t*_min_ were found increased while LVEDP was reduced in I/R rats treated with SEV, and the opposite trends were noted in I/R rats treated with miR‐203 antagomir. Also, miR‐203 antagomir negated the effects of SEV on LVSP, +d*p*/d*t*_max_, −d*p*/d*t*_min_ and LVEDP (Table 3). The expression of oxidative stress markers (MDA, NO, GSH and SOD) in rat myocardial tissues and pro‐inflammatory factors (TNF‐α, IL‐6 and IL‐1β) and myocardial enzymes (CK‐MB, LDH and cTn‐T) in rat serum was measured and as demonstrated in Figure 3B‐G, MDA, NO, TNF‐α, IL‐6, IL‐1β, CK‐MB, LDH and cTn‐T levels in I/R rats were reduced by SEV but GSH and SOD levels were elevated, while treatment miR‐203 antagomir led to the opposite effects and treatment with miR‐203 antagomir counteracted the effects of SEV. The TUNEL assay showed that the apoptotic rate in the myocardial tissues of I/R rats was markedly reduced upon exposure SEV but increased in response to miR‐203 antagomir. As compared to I/R rats treated with SEV alone, the combination treatment with SEV + miR‐203 antagomir augmented the apoptosis in the myocardial tissues of I/R rats (Figure 3H). Furthermore, Western blot analysis showed that the expression levels of Cyt‐c and cleaved caspase‐3 in the myocardial tissues of I/R rats treated with SEV were reduced notably, but enhanced in the myocardial tissues of I/R rats treated with miR‐203 antagomir. Treatment with miR‐203 antagomir normalized the effects of SEV on Cyt‐c and cleaved caspase‐3 expression (Figure 3I). The results of TTC staining indicated decreased infarct size of myocardial tissues in I/R rats treated by SEV, which was abrogated by miR‐203 antagomir treatment, while the infarct size of myocardial tissues in I/R rats was increased upon miR‐203 antagomir treatment (Figure 3J). Taken together, these findings demonstrated that SEV alleviated myocardial I/R injury by reducing oxidative stress, inflammation, apoptosis and infarct size in rats through the up‐regulation of miR‐203.

**TABLE 3:** Effects of SEV on the hemodynamics in rats with myocardial I/R injury through miR‐203

Group	LVSP	LVEDP	+d*p*/d*t*_max_	−d*p*/d*t*_min_
	(mm Hg)	(mm Hg)	(Kmm Hg/s)	(Kmm Hg/s)
I/R	67.38 ± 5.48	31.58 ± 2.69	0.95 ± 0.08	−0.42 ± 0.03
SEV	87.48 ± 7.58*	14.52 ± 1.62*	1.89 ± 0.01*	−0.99 ± 0.11*
antagomir‐NC	62.58 ± 6.98^#^	28.79 ± 2.63^#^	0.77 ± 0.06^#^	−0.64 ± 0.04^#^
miR‐203 antagomir	50.68 ± 4.38^#^	48.37 ± 3.85^#^	0.41 ± 0.02^#^	−0.35 ± 0.01^#^
SEV + miR‐203 antagomir	70.28 ± 6.84^#^	18.48 ± 1.27^#^	1.53 ± 0.01^#^	−0.85 ± 0.07^#^

Note

Results were shown as mean ± SD from three technical repeats (n = 6‐8).

Abbreviations: I/R, ischaemia‐reperfusion; LVEDP, left ventricular end‐diastolic pressure; LVSP, left ventricular systolic pressure; NC, negative control; SEV, sevoflurane.

* *P* < 0.05 vs I/R rats.

^#^ *P* < 0.05 vs I/R rats treated by SEV.

### 3.4 Doublecortin was targeted by miR‐203

**FIGURE 4:**
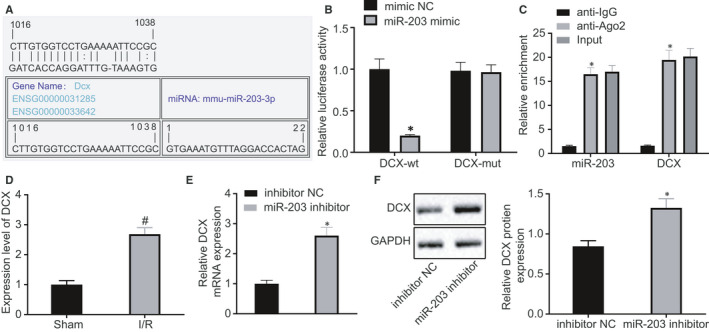
MicroRNA‐203 (miR‐203) targets and down‐regulates doublecortin (DCX). A, The binding site between miR‐203 and DCX predicted by bioinformatics analysis using RNA22. B, The interaction between miR‐203 and DCX verified by dual‐luciferase reporter gene assay. C, The binding of miR‐203 and DCX to Ago2 each, detected by RNA‐binding protein immunoprecipitation assay. D, DCX expression in myocardial tissues of ischaemia‐reperfusion (I/R) rats determined by reverse transcription‐quantitative polymerase chain reaction, normalized to glyceraldehyde‐3‐phosphate dehydrogenase (GAPDH). E, DCX mRNA expression in myocardial tissues of I/R rats treated with miR‐203 inhibitor determined by reverse transcription‐quantitative polymerase chain reaction, normalized to GAPDH. F, DCX protein expression in myocardial tissues of I/R rats treated with miR‐203 inhibitor, normalized to GAPDH, determined by Western blot analysis. *P < 0.05 vs the treatment of inhibitor‐negative control (NC); ^#^ *P* < 0.05 vs sham‐operated rats by one‐way ANOVA, followed by Tukey's post hoc tests. Results were shown as mean ± SD from three technical replicates (n = 6‐8)

To investigate the downstream regulatory mechanisms of miR‐203, bioinformatic analysis was first performed using a web‐based bioinformatic software (https://cm.jefferson.edu/rna22/). The results indicated that miR‐203 sequence contained a binding site for DCX (Figure 4A) and thus suggested that DCX was possibly directly regulated by miR‐203. To validate this hypothesis, a dual‐luciferase reporter gene assay was conducted, and luciferase activity of pMIR‐reporter containing DCX 3ʹUTR WT was found reduced upon cotransfection with miR‐203 mimic (Figure 4B). The RNA‐binding protein immunoprecipitation assay revealed that miR‐203 and DCX were immunoprecipitated at higher levels by Ago2, relative to IgG, which was in agreement with the results of bioinformatic analysis and the dual‐luciferase reporter gene assay (Figure 4C). Doublecortin expression in the myocardial tissues of I/R rats was determined using RT‐qPCR, and the results showed DCX was significantly highly expressed in I/R rats as compared to sham‐operated rats (Figure 4D). In addition, DCX mRNA and protein expression levels in myocardial tissues were elevated upon treatment with miR‐203 inhibitor (Figure 4E, F). Together, these results indicated that miR‐203 was capable of negatively targeting DCX.

### 3.5 MicroRNA‐203 protected rats against myocardial I/R injury by inhibiting DCX

**FIGURE 5:**
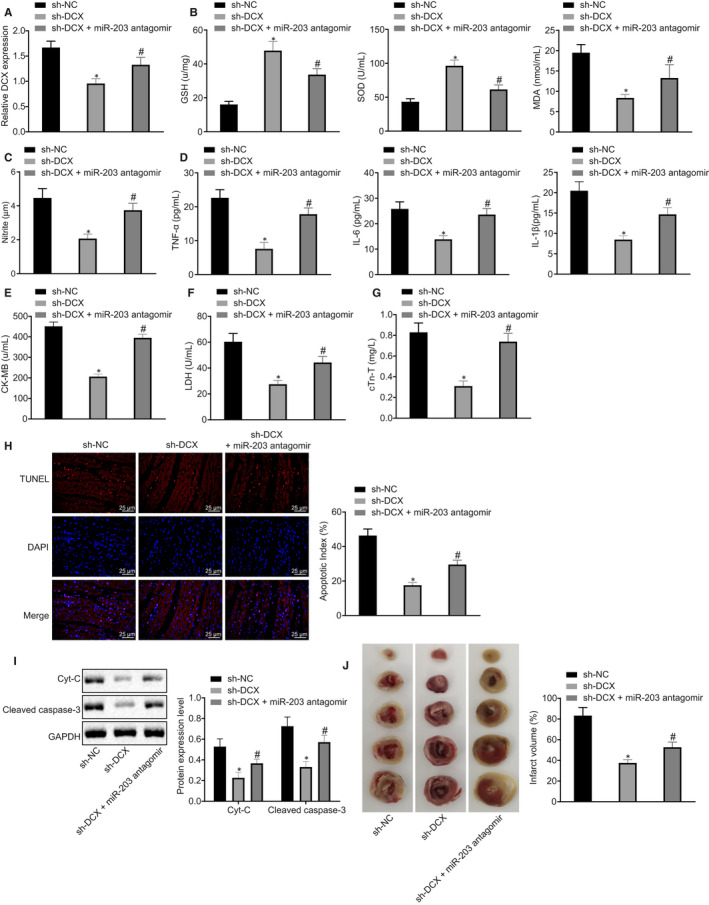
Myocardial ischaemia‐reperfusion (I/R) injury in rats is ameliorated by microRNA‐203 (miR‐203) through down‐regulation of doublecortin (DCX). I/R rats were treated with sh‐negative control (NC), sh‐DCX or sh‐DCX + miR‐203 antagomir. A, DCX expression in myocardial tissues of I/R rats determined by reverse transcription‐quantitative polymerase chain reaction normalized to glyceraldehyde‐3‐phosphate dehydrogenase (GAPDH). B, Levels of malondialdehyde (MDA), glutathione (GSH) and superoxide dismutase (SOD) in myocardial tissues of I/R rats measured by ELISA. C, Nitric oxide level in rat serum determined by nitrite assay. D, Levels of tumour necrosis factor alpha (TNF‐α), interleukin (IL)‐6 and IL‐1β in rat serum measured by ELISA. E, Level of creatine kinase isoenzyme (CK‐MB) in rat serum determined by ELISA. F, Level of LDH in rat serum measured by ELISA. G, Level of cTn‐T in rat serum determined by ELISA. H, Representative myocardial apoptosis determined by TUNEL staining (×400) and apoptotic rates. I, Representative Western blots and their quantification of Cyt‐c and cleaved caspase‐3 in myocardial tissues of I/R rats, normalized to GAPDH. J, Infarct size determined by triphenyltetrazolium chloride staining and infarct volume. *P < 0.05 vs I/R rats treated with sh‐NC, ^#^ *P* < 0.05 vs I/R rats treated with sh‐DCX by one‐way ANOVA, followed by Tukey's post hoc tests. Results were shown as mean ± SD from three technical replicates (n = 6‐8)

To investigate whether miR‐203 affected myocardial oxidative stress and inflammation by down‐regulating DCX, I/R rats were treated with sh‐DCX and miR‐203 antagomir. Reverse transcription‐quantitative polymerase chain reaction results showed that DCX expression in myocardial tissues of I/R rats was diminished upon treatment with sh‐DCX, which was restored by miR‐203 antagomir treatment (Figure 5A). Furthermore, DCX silencing increased LVSP, +d*p*/d*t*_max_ and −d*p*/d*t*_min_ and decreased LVEDP in I/R rats, which was reversed upon treatment with miR‐203 antagomir (Table 4). Next, the expression levels of oxidative stress markers (MDA, NO, GSH and SOD) in rat myocardial tissues, and pro‐inflammatory factors (TNF‐α, IL‐6 and IL‐1β) and myocardial enzymes (CK‐MB, LDH and cTn‐T) in rat serum were investigated, and as depicted in Figure 5B‐G, the levels of MDA, NO, TNF‐α, IL‐6, IL‐1β, CK‐MB, LDH and cTn‐T were reduced while GSH and SOD levels increased upon treatment with sh‐DCX, which was countered by further treatment with miR‐203 antagomir (Figure 5B‐G). The TUNEL assay showed that the apoptotic rate in the myocardial tissues of I/R rats was reduced markedly by sh‐DCX, which was countered by additional treatment with miR‐203 antagomir (Figure 5H). In addition, Western blot analysis showed a notable reduction in expression levels of Cyt‐c and cleaved caspase‐3 in the myocardial tissues of I/R rats treated with sh‐DCX, which was reversed by miR‐203 antagomir treatment (Figure 5I). The results of TTC staining indicated a decreased infarct size in I/R rats treated with sh‐DCX, which was normalized by miR‐203 antagomir treatment (Figure 5J). These results together indicated that miR‐203 alleviated myocardial I/R injury by relieving oxidative stress, inflammation, apoptosis and infarct size in rats through the down‐regulation of DCX.

**TABLE 4:** Effects of miR‐203 on the hemodynamics in rats with myocardial I/R injury through DCX

Group	LVSP	LVEDP	+d*p*/d*t*_max_	−d*p*/d*t*_min_
	(mm Hg)	(mm Hg)	(Kmm Hg/s)	(KmmHg/s)
sh‐NC	51.48 ± 4.83	29.58 ± 3.01	0.61 ± 0.05	−0.51 ± 0.06
sh‐DCX	79.43 ± 6.59*	14.38 ± 1.32*	1.89 ± 1.38*	−1.15 ± 0.14*
sh‐DCX + miR‐203 antagomir	64.36 ± 6.52^#^	26.48 ± 2.41^#^	1.32 ± 1.12^#^	−0.76 ± 0.08^#^

Note

Results were shown as mean ± SD from three technical repeats (n = 6‐8).

Abbreviations: DCX, doublecortin; I/R, ischaemia‐reperfusion; LVEDP, left ventricular end‐diastolic pressure; LVSP, left ventricular systolic pressure; NC, negative control.

* *P* < 0.05 vs I/R rats treated with sh‐NC.

^#^ *P* < 0.05 vs I/R rats treated with sh‐DCX.

## 4 DISCUSSION

Myocardial I/R injury is capable of inducing cardiovascular damage and constitutes the primary pathological presentation in coronary artery disease; thus, it comprises major cause of morbidity and mortality related to coronary occlusion.^16^ Increasing evidence supports a protective role of SEV in myocardial I/R injury.^17^, ^18^ The current study was designed to investigate specific molecular mechanisms underlying SEV‐mediated protection in myocardial I/R injury. Our results demonstrated that SEV positively regulated miR‐203 to reduce DCX expression, thereby repressing oxidative stress, inflammation, apoptosis and infarct area, ultimately alleviating myocardial I/R injury in rats.

Primarily, the levels of MDA, NO, TNF‐α, IL‐6, IL‐1β, CK‐MB, LDH, cTn‐T, Cyt‐c and cleaved caspase‐3 decreased while GSH and SOD increased in rats with myocardial I/R injury upon SEV treatment. These results indicated that SEV was capable of suppressing oxidative stress, inflammation, apoptotic rate and infarct area, all of which attenuated myocardial I/R injury, in broad agreement with the previous finding that SEV is protective against myocardial I/R injury.^4^ Also, SEV post‐conditioning treatment has been noted to alleviate hypoxia‐reoxygenation injury in cardiomyocytes.^19^ Malondialdehyde, SOD and GSH are vital biomarkers of oxidative stress^20^, ^21^, ^22^ and their levels can be considered to reflect the degree of myocardial I/R injury. Similar to our approach, a previous study considered elevated GSH and SOD with decreased MDA as reflective of myocardial I/R injury amelioration by an extract of *Potentilla anserina* L.^23^ Nitric oxide (NO) is an important secondary messenger interplaying with reactive oxygen species and plays significant role in mediating oxidative stress.^24^, ^25^ In particular, NO has been noted as a contributor to myocardial I/R injury.^26^ The down‐regulation of TNF‐α, IL‐1β and IL‐6 has been found to exert an anti‐inflammatory effect on the development of myocardial infarction.^27^ In addition, decreased levels of CK‐MB, LDH and cTn‐T have also been identified as markers of improvement of myocardial ischaemia.^28^ The down‐regulation of Cyt‐c and cleaved caspase‐3 has been previously noted to indicate an inhibition of cell apoptosis and mark the alleviation of myocardial I/R injury.^29^ Aligned with our findings, previous reports have indicated that SEV reduces IL‐6, TNF‐α, MDA, NO, CK‐MB, cTn‐T and cleaved caspase‐3 expression and enhances SOD expression in I/R injury.^30^, ^31^ Together, these data indicate that SEV is promising candidate in the management of myocardial I/R injury owing to its robust cardiac protective function.

We next showed that miR‐203 was poorly expressed in the myocardial tissues of rats with myocardial I/R injury and was up‐regulated by SEV treatment. miRNAs have been documented as valuable biomarkers of myocardial injury.^32^ MicroRNA‐203 up‐regulation has been reported to suppress cardiomyocyte inflammation and myocardial I/R injury and found to be down‐regulated by lncRNA MALAT1.^9^ MicroRNA‐203 is also suggested as potential regulator of neuroinflammation and brain injury.^33^ In a similar finding, miR‐203 was found up‐regulated by SEV treatment in breast cancer cells and thus suggested as a promising target for breast cancer treatment.^10^ Subsequently, we showed miR‐203 suppressed the expression of DCX, which eventually ameliorated myocardial I/R injury. Doublecortin has been shown as a key player in improving cerebral ischaemia.^13^ In a related finding, DCX was found down‐regulated by another miR, miR‐181a in primary neuronal stem cells, which further led to improvement of forebrain ischaemia.^34^ Our data demonstrated that miR‐203 alleviated myocardial I/R injury by relieving oxidative stress, inflammation, apoptosis and infarct size in rats through the down‐regulation of DCX. Doublecortin elevation has also been implicated in brain inflammation and the resultant short‐term memory impairment in rats,^35^ while miR‐203 has been implicated in the alleviation of inflammation, oxidative stress and apoptosis in rats with lung injuries.^36^ Together with the current evidence, DCX silencing induced by miR‐203 may be considered as a potential approach for myocardial I/R injury management. The study must be considered in the light of its limitations. Here, we performed intramyocardial injection of miR‐203 antagomir to achieve specific knockdown of myocardial miR‐203. However, intraperitoneal injection and caudal vein injection of miR‐203 antagomir can also be used for the purpose of miR‐203 knockdown. Comparative knockdown effects produced by intramyocardial injection, intraperitoneal injection and caudal vein injection of miR‐203 antagomir have not been addressed and should be investigated in future research. Moreover, comprehensive molecular mechanisms of SEV‐mediated effects in myocardial I/R injury merit detailed investigation.

Overall, the present study demonstrated that the elevation of miR‐203 by SEV repressed DCX to improve oxidative stress and inflammation, ultimately alleviating myocardial I/R injury in rats. This study validated the cardiac protective effect of SEV, which may be a promising clinically viable agent for myocardial I/R injury treatment.

## CONFLICT OF INTEREST

The authors confirm that there are no conflicts of interest.

## AUTHOR CONTRIBUTION

**Jian Tan**: Conceptualization (equal); Project administration (equal); Resources (equal); Supervision (equal); Writing‐original draft (equal); Writing‐review & editing (equal). **Zhiguo Wu**: Conceptualization (equal); Validation (equal); Visualization (equal); Writing‐original draft (equal); Writing‐review & editing (equal). **Jun Liu**: Data curation (equal); Software (equal); Writing‐review & editing (equal). **Wenting Zhang**: Data curation (equal); Methodology (equal); Writing‐review & editing (equal). **Wanqiu Yuan**: Investigation (equal); Resources (equal); Writing‐review & editing (equal). **Hong Peng**: Formal analysis (equal); Resources (equal); Writing‐review & editing (equal).

## Data Availability

All data supporting the findings of this study are available within the article.

## References

[1] Hausenloy DJ , Yellon DM . Myocardial ischemia‐reperfusion injury: a neglected therapeutic target. J Clin Invest. 2013;123:92‐100. 23281415 10.1172/JCI62874 PMC3533275

[2] Koehn RK . Heterozygosity and growth in marine bivalves: comments on the paper by Zouros, Romero‐Dorey, and Mallet (1988). Evolution. 1990;44:213‐216. 28568217 10.1111/j.1558-5646.1990.tb04292.x

[3] Liu AJ , Pang CX , Liu GQ , et al. Ameliorative effect of sevoflurane on endoplasmic reticulum stress mediates cardioprotection against ischemia‐reperfusion injury. Can J Physiol Pharmacol. 2019;97:345‐351. 29894643 10.1139/cjpp-2018-0016

[4] Dong J , Xu M , Zhang W , et al. Effects of sevoflurane pretreatment on myocardial ischemia‐reperfusion injury through the Akt/hypoxia‐inducible factor 1‐alpha (HIF‐1alpha)/vascular endothelial growth factor (VEGF) signaling pathway. Med Sci Monit. 2019;25:3100‐3107. 31028241 10.12659/MSM.914265 PMC6501450

[5] Zhang Y , Li Y , Han X , et al. Elevated expression of DJ‐1 (encoded by the human PARK7 gene) protects neuronal cells from sevoflurane‐induced neurotoxicity. Cell Stress Chaperones. 2018;23:967‐974. 29728856 10.1007/s12192-018-0904-3 PMC6111095

[6] Xie XJ , Fan DM , Xi K , et al. Suppression of microRNA‐135b‐5p protects against myocardial ischemia/reperfusion injury by activating JAK2/STAT3 signaling pathway in mice during sevoflurane anesthesia. Biosci Rep. 2017;37(3):1–12. 10.1042/BSR20170186 PMC6434087 28522550

[7] Makhdoumi P , Roohbakhsh A , Karimi G . MicroRNAs regulate mitochondrial apoptotic pathway in myocardial ischemia‐reperfusion‐injury. Biomed Pharmacother. 2016;84:1635‐1644. 27829552 10.1016/j.biopha.2016.10.073

[8] Tian SH , Yu DJ , Li ZY , et al. The inhibition of microRNA‐203 on ischemic reperfusion injury after total knee arthroplasty via suppressing MYD88‐mdiated toll‐like receptor signaling pathway. Gene. 2019;697:175‐183. 30772517 10.1016/j.gene.2019.02.030

[9] Wang S , Yu W , Chen J , et al. LncRNA MALAT1 sponges miR‐203 to promote inflammation in myocardial ischemia‐reperfusion injury. Int J Cardiol. 2018;268:245. 30041794 10.1016/j.ijcard.2018.03.085

[10] Liu J , Yang L , Guo X , et al. Sevoflurane suppresses proliferation by upregulating microRNA‐203 in breast cancer cells. Mol Med Rep. 2018;18:455‐460. 29750301 10.3892/mmr.2018.8949

[11] Moslehi M , Ng DCH , Bogoyevitch MA . Dynamic microtubule association of Doublecortin X (DCX) is regulated by its C‐terminus. Sci Rep. 2017;7:5245. 28701724 10.1038/s41598-017-05340-x PMC5507856

[12] Bregere C , Fisch U , Sailer MH , et al. Neonatal hypoxia‐ischemia in rat increases doublecortin concentration in the cerebrospinal fluid. Eur J Neurosci. 2017;46:1758‐1767. 28548285 10.1111/ejn.13612

[13] Goto K , Kutsuna N , Yamashita A , et al. Changes of doublecortin‐immunoreactive cells from the acute phase to chronic phase after transient global brain ischemia in rat cingulate cortex. Adv Exp Med Biol. 2018;1072:69‐75. 30178326 10.1007/978-3-319-91287-5_12

[14] Han J , Zhang JZ , Zhong ZF , et al. Gualou Guizhi decoction promotes neurological functional recovery and neurogenesis following focal cerebral ischemia/reperfusion. Neural Regen Res. 2018;13:1408‐1416. 30106053 10.4103/1673-5374.235296 PMC6108212

[15] Cheng Y , Zhu P , Yang J , et al. Ischaemic preconditioning‐regulated miR‐21 protects heart against ischaemia/reperfusion injury via anti‐apoptosis through its target PDCD4. Cardiovasc Res. 2010;87:431‐439. 20219857 10.1093/cvr/cvq082 PMC2904662

[16] Li X , Liu M , Sun R , et al. Protective approaches against myocardial ischemia reperfusion injury. Exp Ther Med. 2016;12:3823‐3829. 28101167 10.3892/etm.2016.3877 PMC5228114

[17] Wang G , Dai D , Gao H , et al. Sevoflurane alleviates reperfusion arrhythmia by ameliorating TDR and MAPD90 in isolated rat hearts after ischemia‐reperfusion. Anesthesiol Res Pract. 2019;2019:7910930. 31662745 10.1155/2019/7910930 PMC6778868

[18] Huang G , Hao F , Hu X . Downregulation of microRNA‐155 stimulates sevoflurane‐mediated cardioprotection against myocardial ischemia/reperfusion injury by binding to SIRT1 in mice. J Cell Biochem. 2019;120:15494‐15505. 31099069 10.1002/jcb.28816

[19] Yang L , Wu J , Xie P , et al. Sevoflurane postconditioning alleviates hypoxia‐reoxygenation injury of cardiomyocytes by promoting mitochondrial autophagy through the HIF‐1/BNIP3 signaling pathway. PeerJ. 2019;7:e7165. 31275755 10.7717/peerj.7165 PMC6596409

[20] Luan X , Chen H , Qiu H , et al. Association between serum malondialdehyde levels and depression during early methamphetamine withdrawal. Neurosci Lett. 2018;687:22‐25. 30219487 10.1016/j.neulet.2018.09.021

[21] Sujiwattanarat P , Pongsanarakul P , Temsiripong Y , et al. Molecular cloning and characterization of Siamese crocodile (*Crocodylus siamensis*) copper, zinc superoxide dismutase (CSI‐Cu, Zn‐SOD) gene. Comp Biochem Physiol A Mol Integr Physiol. 2016;191:187‐195. 26523498 10.1016/j.cbpa.2015.10.028

[22] Kovacs‐Nolan J , Rupa P , Matsui T , et al. In vitro and ex vivo uptake of glutathione (GSH) across the intestinal epithelium and fate of oral GSH after in vivo supplementation. J Agric Food Chem. 2014;62:9499‐9506. 25198144 10.1021/jf503257w

[23] Zhang L , Jian LL , Li JY , et al. Possible involvement of alpha B‐crystallin in the cardioprotective effect of *n*‐butanol extract of *Potentilla anserina* L. on myocardial ischemia/reperfusion injury in rat. Phytomedicine. 2019;55:320‐329. 30940361 10.1016/j.phymed.2018.10.024

[24] Sahay S , Gupta M . An update on nitric oxide and its benign role in plant responses under metal stress. Nitric Oxide. 2017;67:39‐52. 28456602 10.1016/j.niox.2017.04.011

[25] Sahni S , Hickok JR , Thomas DD . Nitric oxide reduces oxidative stress in cancer cells by forming dinitrosyliron complexes. Nitric Oxide. 2018;76:37‐44. 29522907 10.1016/j.niox.2018.03.003 PMC5916741

[26] Nagasaka Y , Fernandez BO , Steinbicker AU , et al. Pharmacological preconditioning with inhaled nitric oxide (NO): organ‐specific differences in the lifetime of blood and tissue NO metabolites. Nitric Oxide. 2018;80:52‐60. 30114529 10.1016/j.niox.2018.08.006 PMC6198794

[27] Hemalatha KL , Stanely Mainzen Prince P . Anti‐inflammatory and anti‐thrombotic effects of zingerone in a rat model of myocardial infarction. Eur J Pharmacol. 2016;791:595‐602. 27568839 10.1016/j.ejphar.2016.08.023

[28] Wang XP , Wang PF , Bai JQ , et al. Investigating the effects and possible mechanisms of danshen‐honghua herb pair on acute myocardial ischemia induced by isoproterenol in rats. Biomed Pharmacother. 2019;118:109268. 31545239 10.1016/j.biopha.2019.109268

[29] Song L , Gao LN , Wang J , et al. Stromal cell‐derived factor‐1alpha alleviates calcium‐sensing receptor activation‐mediated ischemia/reperfusion injury by inhibiting caspase‐3/caspase‐9‐induced cell apoptosis in rat free flaps. Biomed Res Int. 2018;2018:8945850. 29568770 10.1155/2018/8945850 PMC5820583

[30] Xu Z , Yu J , Wu J , et al. The effects of two anesthetics, propofol and sevoflurane, on liver ischemia/reperfusion injury. Cell Physiol Biochem. 2016;38:1631‐1642. 27119513 10.1159/000443103

[31] Zhang J , Zhang J , Yu P , et al. Remote ischaemic preconditioning and sevoflurane postconditioning synergistically protect rats from myocardial injury induced by ischemia and reperfusion partly via inhibition TLR4/MyD88/NF‐kappaB signaling pathway. Cell Physiol Biochem. 2017;41:22‐32. 28135708 10.1159/000455815

[32] Hortmann M , Walter JE , Benning L , et al. Droplet digital PCR of serum miR‐499, miR‐21 and miR‐208a for the detection of functionally relevant coronary artery disease. Int J Cardiol. 2019;275:129‐135. 30126654 10.1016/j.ijcard.2018.08.031

[33] Yang Z , Zhong L , Zhong S , et al. miR‐203 protects microglia mediated brain injury by regulating inflammatory responses via feedback to MyD88 in ischemia. Mol Immunol. 2015;65:293‐301. 25723469 10.1016/j.molimm.2015.01.019

[34] Griffiths BB , Ouyang YB , Xu L , et al. Postinjury inhibition of miR‐181a promotes restoration of hippocampal CA1 neurons after transient forebrain ischemia in rats. eneuro. 2019;6(4):1–11. 10.1523/ENEURO.0002-19.2019 PMC6727148 31427401

[35] Kim SE , Ko IG , Park CY , et al. Treadmill and wheel exercise alleviate lipopolysaccharide‐induced short‐term memory impairment by enhancing neuronal maturation in rats. Mol Med Rep. 2013;7:31‐36. 23128607 10.3892/mmr.2012.1160

[36] Ling L , Lu HT , Wang HF , et al. MicroRNA‐203 acts as a potent suppressor in septic shock by alleviating lung injury via inhibition of VNN1. Kidney Blood Press Res. 2019;44:565‐582. 31340209 10.1159/000500484

